# Transient otoacoustic emissions with contralateral suppression findings in COVID-19 patients

**DOI:** 10.1186/s43163-022-00231-z

**Published:** 2022-03-21

**Authors:** Meliha Basoz, Nida Tas, Ozge Gedik, Sumeyye Ozdemir, Fadlullah Aksoy

**Affiliations:** 1 Health Sciences Faculty, Audiology Department, Bezmialem Vakif University, Fatih, Istanbul, Turkey; 2 Department of Otorhinolaryngology, Bezmialem Vakif University, Fatih, Istanbul, Turkey

**Keywords:** COVID-19, Transient otoacoustic emissions with contralateral suppression, Efferent auditory system, Auditory system, Hearing

## Abstract

**Objective:**

The virus called SARS-CoV-2, which is known as the first epidemic of the twenty-first century, is known to affect the central and peripheral nervous system. In the literature, complaints of sudden hearing loss, tinnitus, and vertigo have been reported in the patients.

The aim of this study is to objectively reveal the effect of the coronavirus disease 2019 on the efferent auditory system.

**Methods:**

Twenty-three participants, who had the coronavirus disease 2019, were included in the study group, while 20 healthy participants were included in the control group. The test of transient otoacoustic emissions with contralateral suppression was applied to individuals who had normal audiological and immitansmetric evaluations findings.

**Results:**

In audiological evaluation, a significant difference was observed between the groups in the 125–500 Hz low frequency range and 4 kHz–12.5 kHz high frequency range. In the test of TEOAE and TEOAE with contralateral suppression, a significant difference was observed between the groups at 4 kHz.

**Conclusion:**

The effect of the coronavirus disease 2019 on the peripheral hearing system has been shown. Its effect on the efferent hearing system in the high frequency region has been revealed.

**Supplementary Information:**

The online version contains supplementary material available at 10.1186/s43163-022-00231-z.

## Background

Known as the first epidemic of the twenty-first century, the coronavirus disease 2019 (COVID-19), caused by the virus called SARS-CoV-2, first appeared in Wuhan, China, in 2019 and has become a global epidemic in the course of time [1]. The disease has caused 2.64 million deaths by March 2021 [2]. It has been reported that the disease can be symptomatic and asymptomatic; its effects have differed by race, gender, and age [1].

Although the data have showed that the most common symptoms are fatigue, cough, sore throat, fever, pneumonia, and anosmia, neurological symptoms such as stroke, epileptic seizure, and encephalitis involving the central and peripheral nervous system and skeletal muscles have also been observed in patients [1, 2]. Otological symptoms have been observed more frequently in younger age groups and women. The symptoms have been mostly in the form of cough, sore throat, and anosmia [3]. Although the effects of the virus on hearing and balance nerve pathways are not exactly known, the complaints of sudden hearing loss, tinnitus, and vertigo seen in patients without major symptoms have strengthened this possibility [3]. There are also studies showing that tinnitus emerging after COVID-19 is caused by social isolation and stress [4]. Although studies have revealed that hearing loss in individuals with COVID-19 is mostly the sensorineural type, there are also patients with conductive hearing loss [5]. Hearing losses have been generally observed at high frequencies and rarely at low frequencies [4, 6]. The effects of COVID-19 on cochlear structures have been examined and decreased transient-evoked otoacoustic emissions (TEOAE) and distortion product otoacoustic emissions (DPOAE) amplitudes have been obtained in patients [4].

The hearing system is divided into two as peripheral and central parts. The peripheral auditory system includes outer, middle, and inner ear structures; the central hearing system covers afferent and efferent nerve pathways. The afferent pathway contains nerve fibers extending from the auditory nerve to the cortex; the efferent nervous system includes the olivocochlear system [7]. The lateral olivocochlear efferent system innervates on the dendrites of auditory nerve fibers under inner hair cells (IHCs); the medial olivocochlear efferent system innervates the outer auditory cells [8]. The efferent system has functions such as improving the signal-to-noise ratio, protecting auditory system from acoustic trauma and decreasing gains in the presence of contralateral stimulation [9, 10]. Medial olivocochlear activity is evaluated by contralateral stimulation with suppression in OAE test [10]. The MOC reflex provides protection against receptor damage during acoustic exposure by reducing the gain of the outer hair cells’ mechanical response to stimuli [9]. When analyzing the TEOAE test with suppression, the difference between the values in the presence and absence of noise is calculated. Loss of contralateral suppression is seen in the presence of brain stem lesions, dyslexia, auditory processing disorder, autoimmune, and metabolic diseases [7, 10].

Some studies have revealed that there is also decreased suppression in patients with vestibular neuronitis caused by herpes simplex virus [11]. Studies have made us think that SARS-CoV-2 may affect the efferent system in patients and cause a decrease in contralateral suppression. Therefore, it has leaded to a more detailed efferent system examination in patients with COVID-19.

## Methods

This study was conducted in Bezmialem Foundation University Audiology Clinic, and approval was obtained from Bezmialem Foundation University Ethics Committee. Number of the ethics committee: 54022451-050.01.04-.

### Participants

The study included 23 [18 female, 5 male] patients aged between 20 and 40 years diagnosed with COVID-19 at Bezmialem Foundation University at least 1 month prior to the examination and without auditory, vestibular, and balance complaints before COVID-19. The patient group had no history of noise exposure, head trauma, usage of ototoxic drugs, and otologic, central, systemic, and metabolic diseases: 20 [16 female, 4 male] normal volunteers and students of the audiology department with normal hearing (pure tone hearing thresholds at 20 dB HL and above at 0.125–8 kHz and having ipsilateral and contralateral acoustic reflexes at bilateral 0.5, 1, 2, and 4 kHz and bilateral type A tympanogram as a result of immitansmetric evaluation) and without history of vertigo and balance complaints. Verbal and written consent were obtained from each participant of the study. Participants from both groups have not been vaccinated against COVID-19 at the time of the examination.

### Audiometric and immitansmetric tests

In order to verify bilateral normal middle ear function, all participants underwent immitansmetric evaluation at 226 Hz and acoustic reflex testing at 500–1000–2000–4000 Hz with Tympstar Pro device of GSI. Air conduction thresholds of 125–8000 Hz and bone conduction thresholds of 250–4000 Hz were evaluated. Extended high frequency audiometry from 10 to 16 kHz was determined. In speech audiometry, the speech reception thresholds (SRT), the most comfortable levels (MCL), and the uncomfortable loudness levels (UCL) were determined, and discrimination scores (SD) scores were calculated.

### Otoacoustic emissions test

Transient-evoked otoacoustic emissions (TEOAE) measurement was recorded with linear click stimulus at 75 ± 4 dB peSPL at frequencies of 1000, 1400, 2000, 2800, and 4000 Hz. Reproducibility [70% and above], stability [80% and above], and stimulus intensity [75 ± 4 dB peSPL and SNR > 3 dB] parameters were followed. Distortion product otoacoustic emissions (DPOAE) measurement was recorded with non-linear click stimulus with f2/f1 rate 1.22, f1 intensity [L1 = 65] and f2 intensity [L2 = 55] L1–L2 10 dB at frequencies of 1000, 1400, 2000, 2800, and 4000 Hz. Reproducibility [70% and above], stability [80% and above], and SNR > 6 dB parameters were followed.

### Contralateral suppression test with otoacoustic emissions

ILO 292 Echoport USB II device was used for contralateral suppression test with otoacoustic emissions. Separately for the right and left ears, it was recorded with linear click stimulus at 75 ± 4 dB peSPL in the presence of broadband white noise provided with contralateral 2 s intervals with 60 dB SPL intensity at frequencies of 1000, 1400, 2000, 2800, and 4000 Hz. The followed parameters were as follows: reproducibility [70% and above], stability [80% and above], stimulus intensity [75 ± 4 dB peSPL], contralateral stimulus intensity [60 dB SPL] sweep [260], and broadband white noise as contralateral noise type. The presence of suppression was decided if there was at least 1 dB amplitude decrease in at least 3 frequencies.

### Statistical analysis

All statistical data were collected and analyzed using IBM SPSS Statistics 22.0 program at *α* = 0.05 significance level. The distribution of the data was examined using the Shapiro-Wilk test. *T* test or Mann-Whitney *U* test was used in comparing the two groups. Descriptive statistics of the data are given as mean ∓ S. Deviation median [min-max] and *n* [%].

## Results

In the study, there were 23 patients and 20 normal individuals between the ages of 20–40. The mean age of the control group was 29.3, the mean age of the patient group was 31, and there was no significant difference between the two groups (Table 1). There was no significant relationship between gender and the group [*p* = 0.52].

**Table 1:** Average age of the groups

	Control	Patient	*p* value
AGE	29.3±7.6	31.0±7.8	0.298

### Audiological tests results

**Fig. 1:**
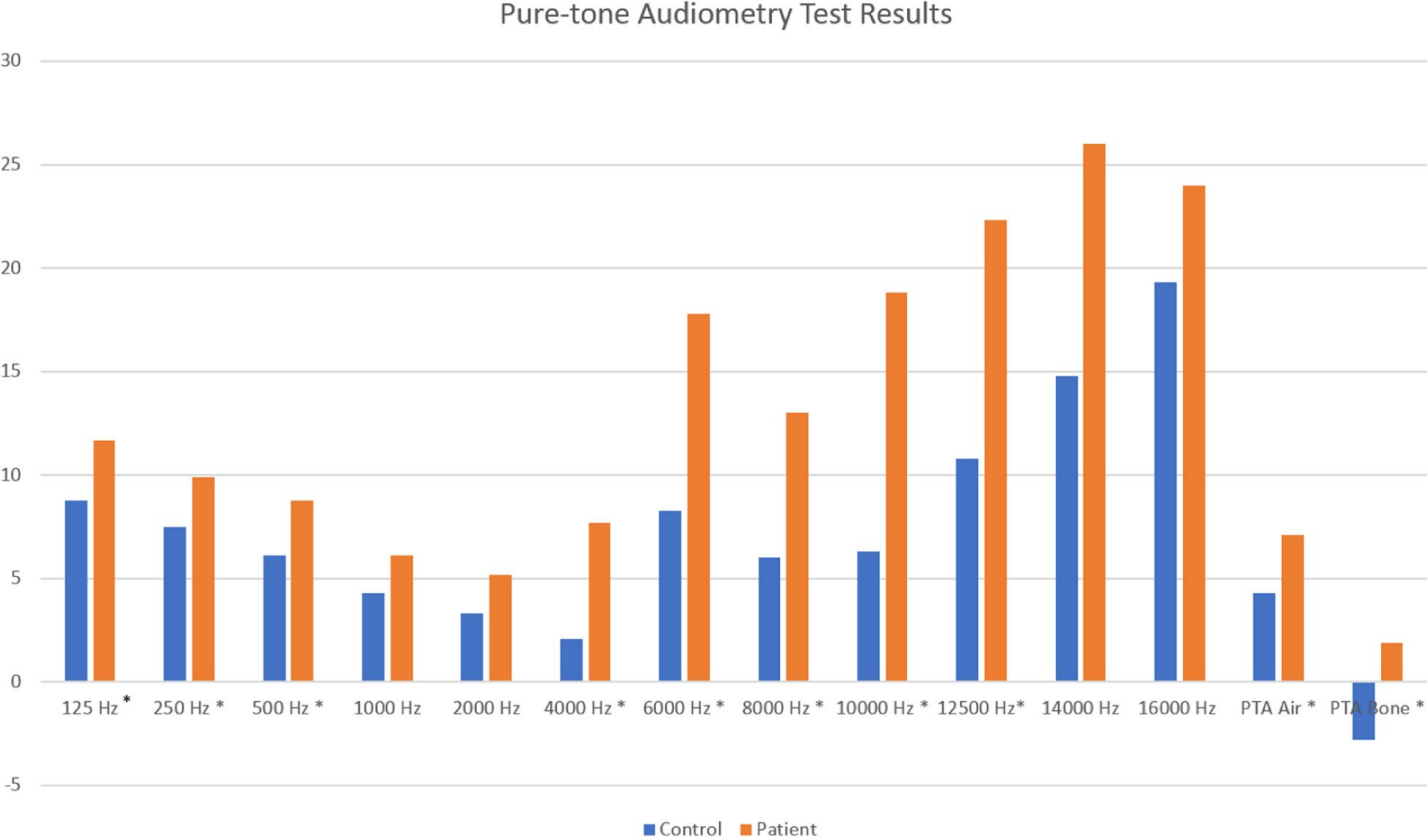
Audiometric results of groups. Significant difference was observed in columns with asterisks [*] [*p* < 0.05]

Air conduction thresholds of 125, 250, 500, 4000, 6000, 8000, 10,000, and 12,500 Hz, air and bone PTAs of the patient group were found significantly higher than the control group [*p* < 0.05] (Fig. 1).

When the speech tests of the participants were examined, the SRTs of the patient group were significantly higher [*p* = 0.000], while a significant decrease was not obtained in the speech discrimination scores [*p* = 0.804] (Table 2).

**Table 2:** Speech audiometry results of the groups

	Control		Patient		*p* value
	Mean	Standard deviation	Mean	Standard deviation	
**SRT**	**8.4**	**4.4**	**13.4**	**4.8**	0.000*
SD	99	2.2	98.9	2.6	0.804

*SRT* speech reception thresholds, *SD* discrimination scores

**Fig. 2:**
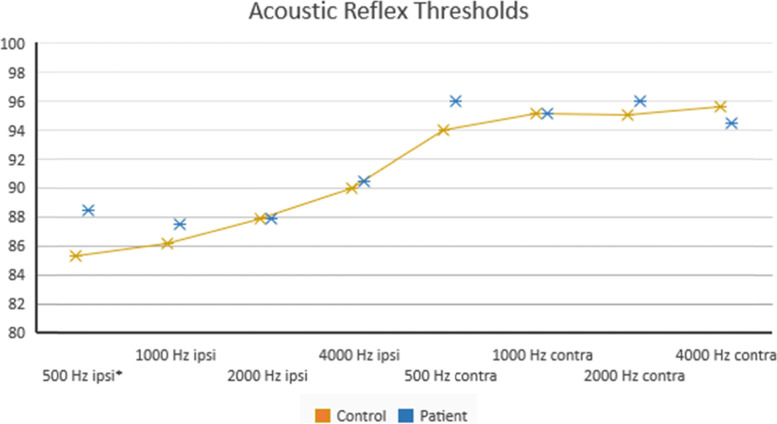
Acoustic reflex thresholds of groups. In the patient group, the 500 Hz ipsilateral acoustic reflex thresholds were significantly higher [*p* = 0.024, *p* < 0.05]

In the acoustic reflex evaluations, only 500 Hz ipsilateral reflex thresholds of the patient group were found significantly higher [*p* = 0.024] (Fig. 2).

### Otoacoustic emissions test results

TEOAE (TE) and DPOAE (DP) tests were applied to the groups to evaluate the cochlear function. In the evaluation, it was seen that there was no significant difference in DPOAE and TEOAE-1000 Hz (TE1), TEOAE-1400 Hz (TE1.4), TEOAE-2000 Hz (TE2), and TEOAE-2800 Hz (TE2.8) values between the groups. However, there was a significant decrease in the TEOAE-4000 Hz (TE4) value in the patient group [*p* = 0.005] (Table 3). The results showed that high frequency region of the cochlea in the patient group was affected.

**Table 3:** Otoacoustic emissions test results in groups

	Control		Patient		*p* value
	Mean	Standard deviation	Mean	Standard deviation	
TE1	7.7	6.2	7.8	6.7	0.930
TE1.4	10.9	6.2	11.0	6.2	0.983
TE2	8.4	5.2	8.1	7.0	0.825
TE2.8	9.0	5.0	6.9	5.1	0.059
**TE4**	**7.7**	**5.5**	**4.2**	**5.8**	0.005*
DP1	− .4	7.8	.0	7.1	0.812
DP1.4	8.3	7.9	9.0	20.7	0.191
DP2	10.1	6.9	11.8	8.3	0.101
DP2.8	10.3	6.6	9.5	7.6	0.515
DP4	12.4	7.9	13.1	6.4	0.673

*TEOAE* transient evoked otoacoustic emissions, *DP* distortion product otoacoustic emissions, *TE1* TEOAE-1000 Hz, *TE1.4* TEOAE-1400 Hz, *TE2* TEOAE-2000 Hz, *TE2.8* TEOAE-2800 Hz, *TE4* TEOAE-4000 Hz, *DP1* DPOAE-1000 Hz, *DP1.4* DPOAE-1400 Hz, *DP2* DPOAE-2000 Hz, *DP2.8* DPOAE-2800 Hz, *DP4* DPOAE-4000 Hz

### Contralateral suppression test with transient otoacoustic emissions

When the mean suppression values between the groups were compared, it was observed that the mean contralateral suppression test with transient otoacoustic emissions-4000 Hz was significantly lower in the control group compared to the patient group (Table 4).

**Table 4:** Mean contralateral suppression with transient otoacoustic emissions in the groups

Frequency	Control		Patient		*p* value
	Mean	Standard deviation	Mean	Standard deviation	
1 kHz	− 1.1	3.1	− 1.3	4.1	0.856
1.4 kHz	− 0.6	2.5	− 0.5	2.9	0.757
2 kHz	− 0.6	2.3	− 0.3	2.3	0.539
2.8 kHz	− 0.2	1.9	− 0.7	2.6	0.326
**4 kHz**	− **0.1**	**1.7**	**0.6**	**1.5**	0.044*

Forty ears in the control group and 46 ears in the patient group were compared. It was found that 30% of the ears of the control group and 34.8% of the ears of the patient group had suppression. There was no significant relationship between the groups [*p* = 0.40].

## Discussion

Pneumonia cases, which had unknown etiology and were seen in Wuhan, China, in December 2019, prompted people to make researches. COVID-19 was identified on January 6, 2020, and was accepted as a global epidemic [12]. The disease causes respiratory system problems, cardiomyopathy, and acute cerebrovascular ischemic attacks as well as neurological and gastrointestinal problems [2, 13].

Studies have also revealed that the disease can cause various auditory symptoms such as hearing loss and tinnitus [3, 4]. Based on these findings, we investigated whether patient with COVID-19 disease have reduced MOC efferent activity compared with control subjects, as measured by the contralateral suppression of TEOAEs.

Chern et al. [14] observed a mixed hearing loss in one ear and sensorineural type hearing loss in the other ear. Lang et al. [15] found sensorineural hearing loss that showed decrease towards high frequencies unilaterally. Almufarri et al. [12] found that one of the patients had sensorineural hearing loss, while another patient had conductive hearing loss due to middle ear inflammation. Although the general opinion suggests that sensorineural hearing loss is seen at high frequencies, Chirakkal et al. [4] and Swain et al. [16] found that the sensorineural hearing loss observed in patients was at low frequencies. In our study, 23 patients and 20 healthy individuals were compared; air and bone conduction thresholds were compared in the patients at 125–8000 Hz. A significant decrease was obtained in the air and bone conduction PTAs of the patient group and at 125–250–500–4000–6000–8000 Hz. The data we obtained in the conventional audiological evaluation are compatible with the literature, and a decrease was found at high and low frequencies. In the high-frequency audiometry, a significant decrease was obtained in the patient group at 10,000–12,500 Hz.

In speech audiometry, a significant decrease was obtained in SRT levels in the patient group, and the result is also compatible with the literature [17].

Although it is known that viruses generally cause sensorineural hearing loss by affecting cells in the cortex, the effects of COVID-19 on the auditory system are not clear yet [18]. In previous studies, inner ear was evaluated with TEOAE and DPOAE [4, 12, 16]. Chirakkal et al. (2020) could not obtain TEOAE responses at low frequencies, while Almufarrij et al. [12] observed a decrease in amplitudes at high frequencies. In our study, a significant decrease was observed in TEOAE amplitudes at 4000 Hz in the patient group, and no significant difference was detected at low frequencies and DPOAE responses. We think that this is related to the fact that TEOAE is more sensitive to cochlear damage than DPOAE [19]. In the study of Gedik et al. [17] low signal-to-noise ratio was found in TEOAE at 4 kHz in the patient group. The study supported our present findings. In some studies examining the effects of ototoxic drugs and noise on hearing, it was observed that the outer hair cells in the basal region of the cochlea were affected more than the outer hair cells in the apex. This was explained by the intrinsic sensitivity of hair cells in the basal region [20]. Likewise, in our study, we think that the high frequency hearing loss caused by the COVID-19 virus is related to the intrinsic sensitivity of the hair cells in the basal region.

In previous studies, vestibular neuronitis caused by the herpes simplex virus, efferent system affection has been observed in patients [11]. In a study conducted by Celik et al. [21], 37 infants, whose mothers had COVID-19 during pregnancy, and 36 infants, whose mothers were healthy, were compared. In infants, whose mothers had COVID-19, contralateral suppression was found to be significantly lower at 2000, 3000, and 4000 Hz [21]. This situation made us think that COVID-19 may also affect the efferent system; therefore, the efferent system was examined by otoacoustic emissions with contralateral suppression. The data of the study suggested that there was a significant increase in suppression values of 4000 Hz in the individuals with COVID-19 (mean = 0.6) compared to the control group (mean = − 0.1) [*p* = 0.044]. Although this suggests that efferent system affection is mostly observed in the high-frequency region, our findings support the study conducted by Celik et al. [21] When the ears in the control and study groups were compared, while 30% of the ears in the control group had suppression, 34% of the ears in the study group had suppression, and no significant difference was observed between the groups.

## Conclusion

In our study, in addition to the negative effect of COVID-19 on the peripheral auditory system, it has been revealed that efferent auditory system affection starts from the high-frequency region.

## Limitations

Because the patient group did not have pre-COVID-19 results, the patient group was compared with the normal group. This was accepted as the limitation of the study.

## Supplementary Information

**Additional file 1.**

## Data Availability

Added to supplementary file.

## Abbreviations

COVID-19: Coronavirus disease
OAE: Otoacoustic emissions
TEOAE: Transient evoked otoacoustic emissions
DPOAE: Distortion product otoacoustic emissions
HL: Hearing level
SPL: Sound pressure level
SRT: Speech reception thresholds
MCL: Most comfortable levels
UCL: Uncomfortable loudness levels
SD: Discrimination scores
TE1: Transient evoked otoacoustic emissions-1000 Hz
TE1.4: Transient evoked otoacoustic emissions-1400 Hz
TE2: Transient evoked otoacoustic emissions-2000 Hz
TE2.8: Transient evoked otoacoustic emissions-2800 Hz
TE4: Transient evoked otoacoustic emissions-4000 Hz
DP1: Distortion product otoacoustic emissions-1000 Hz
DP1.4: Distortion product otoacoustic emissions-1400 Hz
DP2: Distortion product otoacoustic emissions-2000 Hz
DP2.8: Distortion product otoacoustic emissions-2800 Hz
DP4: Distortion product otoacoustic emissions-4000 Hz
